# Correlation between Platelet Gelsolin and Platelet Activation Level in Acute Myocardial Infarction Rats and Intervention Effect of Effective Components of Chuanxiong Rhizome and Red Peony Root

**DOI:** 10.1155/2013/985746

**Published:** 2013-03-07

**Authors:** Yue Liu, Huijun Yin, Yuerong Jiang, Mei Xue, Chunyu Guo, Dazhuo Shi, Keji Chen

**Affiliations:** Cardiovascular Disease Centre, Xiyuan Hospital, China Academy of Chinese Medical Sciences, Beijing 100091, China

## Abstract

The biological role of platelet gelsolin in platelet activation of acute myocardial infarction is not defined. In order to provide a potential new antiplatelet target for Chinese medicine and to elucidate the contribution of Xiongshao capsule, the effective components of Chuanxiong rhizome and red peony root, in this study, we randomly allocated Sprague Dawley rats to left anterior descending coronary artery ligation or sham surgery and different drug prophylaxis as control. We found that gelsolin is highly expressed in platelet rich plasma and lowly expressed in platelet poor plasma, accompanied by the high platelet activation level in model rats; plasma actin filaments and mean fluorescence intensity (MFI) of platelet calcium ion increased and plasma vitamin D binding protein decreased in model rats. Xiongshao capsule could inhibit the gelsolin expression in platelet rich plasma and ischemic heart tissue simultaneously and reduce the level of plasma F-actin and MFI of platelet calcium ion. Our study concludes that platelet gelsolin is an important contributor to platelet activation, and platelet gelsolin inhibition may form a novel target for antiplatelet therapy. Xiongshao capsule may be a promising Chinese medicine drug for antiplatelet and aspirin-like cardioprotection effect.

## 1. Introduction

Despite recent medical advances, cardiovascular diseases remain the predominant cause of morbidity and mortality all over the world [1, 2]. Rupture of atherosclerotic plaque and the ensuing thrombotic changes are the triggers for acute coronary event. Platelet activation and aggregation play crucial roles in this process of atherothrombosis. The emergence of antiplatelet drug is the milestone of prevention and therapy of cardiovascular disease and provides the primary therapeutic strategy to combat cardiovascular diseases. The proper application of antiplatelet drug in reducing the mortality and morbidity of acute myocardial infarction successfully has been verified by a large number of large-scale clinical trials [3]. Antiplatelet drug, such as aspirin, now is recommended for the secondary prevention of cardiovascular disease (CVD) in patients with CVD because it decreases the risk of CVD events and mortality in clinical trials of men and women with CVD [4]. But many clinical problems arose along with the wide range of application of antiplatelet drugs (such as aspirin and clopidogrel, etc.) during the past 10 years [5, 6]. Despite their proven benefits, recurrent cardiovascular events still occur in those taking antiplatelet drugs. This has led to the concept of “antiplatelet drug resistance,” most commonly aspirin or clopidogrel resistance. The latest research shows that aspirin prophylaxis in people without prior CVD does not lead to reductions in cardiovascular death, for the benefits are further offset by clinically important bleeding events [7], which limit the clinical practice of antiplatelet drugs widely. These phenomena suggest that other pathways capable of stimulating platelet activation may exist and provide an impetus for developing new antiplatelet drugs which possess higher efficacy and fewer adverse effects. 

Proteomics technology has been successfully applied to platelet research during the past 5 years, contributing to the emerging field of platelet proteomics which led to the identification of a considerable amount of novel platelet proteins, many of which have been further studied at their functional level [8]. Our previous work [9] indicated that platelet gelsolin is the main platelet differential functional protein between patients of coronary heart disease and healthy people by platelet proteomics. Studies have also shown that platelet gelsolin is highly expressed in patients with acute coronary syndrome (ACS) and the blood-stasis syndrome (BSS) of traditional Chinese medicine (TCM) [10, 11]. Gelsolin is known to have one of the key roles in extracellular actin-scavenger system (EASS) [12], but the biological role of platelet gelsolin in platelet activation of acute myocardial infarction (AMI) is unclear. On the prevention of atherosclerosis or vulnerable plaque, Chinese medicine and Western medicine agree on stabling plaque and promoting blood circulation. Based on the agreed thoughts of the Eastern and Western worlds, the application of Chinese herbs for activating blood circulation (ABC herbs) has valuable significance in the exploration of reducing the risk of cardiovascular event [13, 14]. Chuanxiong rhizome and Red peony root are the two classical ABC herbs in China and have been used for thousands of years in the prevention and treatment of CVD. Xiongshao capsule (XSC) is a patent drug in China and is composed of effective components of Chuanxiong rhizome and Red peony root. Our previous studies have showed that paeoniflorin, ferulic acid and total phenolic acid are the major active principles of the water extract from Xiongshao capsule [15, 16]. Clinical studies indicated that XSC can effectively prevent restenosis after percutaneous coronary intervention (PCI) [17], but the antiplatelet target of XSC is not defined.

In the present study, we used AMI as a disease model to investigate the correlation between platelet gelsolin and platelet activation level in rat model of AMI and the prophylaxis mechanism of XSC *in vivo*.

## 2. Materials and Methods

### 2.1. Drug and Reagents

Xiongshao Capsule (XSC), which contained paeoniflorin (more than or equal to 28 mg each capsule), ferulate (more than or equal to 3.5 mg each capsule), and total phenolic acid (more than or equal to 34 mg each capsule), 0.25 g per capsule, were provided by Beijing International Institute of Biological Products (batch no. 200091, Beijing, China); aspirin, 0.1 g per capsule, was purchased from Bayer HealthCare Manufacturing (batch no. BJ01653, Beijing, China); verapamil, 0.04 g per tablet, was purchased from the Central Pharmaceutical Co., Ltd (batch no. 100402, Tianjing, China). All the drugs were dissolved in pure water before use.

Fluo-3AM was purchased from Sigma (St Louis, MO, USA); rabbit anti-gelsolin polyclonal antibody was purchased from Abcam (San Francisco, USA); mouse anti-*β*-actin monoclonal antibody was purchased from Sigma (St Louis, MO, USA); FITC-Phalloidin was purchased from Sigma (St Louis, MO, USA); enzyme-linked immunosorbent assay (ELISA) kit of P-selectin, gelsolin, F-actin, vitamin D binding protein (VDBP), CK-MB, cTnI, TXB_2_, and COX-1 were purchased from Huamei Biological Technology Company (Wuhan, Hubei province, China).

### 2.2. Animal Grouping and Treatment

Sprague Dawley (SD) rats (male, weight 220–250 g, *n* = 90) were obtained from Beijing University Laboratory Animal Center (the animal certificate No: SCXK (Jing) 2006–0009). The rats were housed in humidity-controlled (55 ± 5)% rooms at (22 ± 2)°C with a 12 h on/12 h off light cycle. The animals were maintained with free access to standard diet and tap water.

After one week of adaptive feeding, we randomly allocated the SD rats into six groups of 15 rats each as follows: Model group, Sham group, Aspirin group, Xscd group (the high dose group), Xscx group (the low doses group), and Verapamil group. Aspirin 40 mg/kg/day, verapamil 4 mg·kg^−1^ d^−1^, and XSC 390 mg/kg/day, 195 mg/kg/day per gavage for 3 consecutive weeks were administrated to the aspirin, verapamil, Xscd and Xscx groups respectively. Rats in the Sham and Model groups received the same volume of distilled water, per gavage for 3 weeks. After 3 weeks, myocardial infarction (MI) model was created in rats by ligating the left anterior descending coronary artery (LAD) as described before [18]. The Animal Care and Use Committee of Xiyuan hospital approved the experimental protocol.

### 2.3. Sample Preparation

After 3 hours of ligation, all the rats were killed after anesthesia by intraperitoneal injection of 20% urethane (0.5 mL/100 g). Fresh blood (10 mL) was drawn from the abdominal aorta and collected into vacutainer tubes containing acid citrate dextrose (ACD) 9% v/v (trisodium citrate 22.0 g/L, citric acid 8.0 g/L, dextrose 24.5 g/L) as anticoagulant. The initial 2 mL of blood was discarded to avoid spontaneous platelet activation. The blood was centrifuged for 10 min at 150 ×g at room temperature to obtain platelet-rich plasma (PRP) and the remaining blood centrifuged for 20 min at 800 ×g to obtain platelet poor plasma (PPP). 

Ischemic heart tissue was taken after blood collection and preserved at −80°C for detection of gelsolin expression by western blotting.

### 2.4. Enzyme-Linked Immunosorbent Assay Analysis

The concentration of PRP and PPP of gelsolin, plasma F-actin, VDBP, CK-MB, cTnI, TXB_2_, COX-1 were determined by enzyme-linked immunoadsorbent assay (ELISA), as per the manufacturer's instructions. The absorbance was measured at 450 nm in an ELISA reader.

### 2.5. Western Blotting Analysis

The level of gelsolin in ischemic heart tissues was determined by Western blot analysis according to the standard procedure as described previously [19]. *β*-actin was used as a loading control.

### 2.6. Detection of MFI of Platelet Calcium Ion

Platelet-rich plasma was prepared and incubated with 4 *μ*mol/L Fluo-3-AM (Sigma, Saint Louis, MO, USA) at 37°C for 40 min. The calcium concentration of platelets was determined using flow cytometry to measure the mean fluorescence intensity (MFI), as previously described [20].

### 2.7. Statistical Analysis

Data are presented as mean ± SD. The SPSS Statistics 11.0 package was utilized to analyze the data. Differences among groups were analyzed using the one-way analysis of variance (ANOVA), followed by multiple comparisons by Least-Significant Difference (LSD) test. Spearman's correlation coefficients were calculated to study the relations between gelsolin concentration in PRP and plasma P-selectin level. Differences between groups were at *P* < 0.05.

## 3. Results 

### 3.1. General Condition

All the rats in the different groups survived and exhibited normal physical appearance and behavior during the gavage period of different drugs. The outcome among the different groups after ligation of LAD is presented in Table 1.

### 3.2. XSC Reduces the Concentration of Myocardial Injury Markers

We chose CK-MB and cTnI as the myocardial injury markers in rats with acute myocardial infarction (AMI). Compared with the Sham group, the concentration of CK-MB and cTnI of Model group increased significantly after ligation of LAD for 3 hours (*P* < 0.01). The high dose of XSC (390 mg/kg/day) can reduce the concentration of CK-MB and cTnI markedly (*P* < 0.05); this has similar effect with aspirin *in vivo* (see Table 2).

### 3.3. XSC Inhibits the Platelet Activation Level

**Figure 1:**
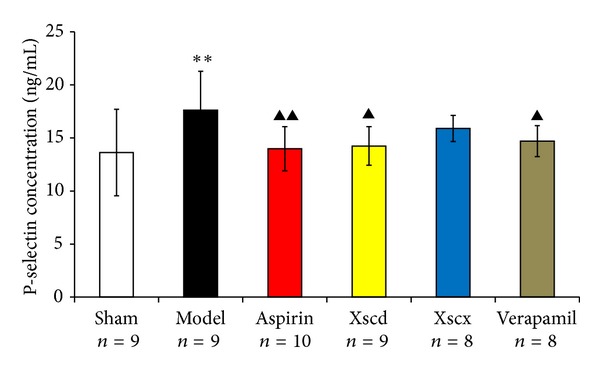
Effect of Xiongshao Capsule (XSC) on P-selectin concentration of AMI rats. **P < 0.01 compared to Sham group, and ^▴^ *P* < 0.05 or ^▴▴^ *P* < 0.01 compared to Model group.

We choose the plasma P-selectin as the marker of platelet activation level. Compared with Sham group, the plasma P-selectin concentration of the Model group increased significantly after ligation of LAD for 3 hours (*P* < 0.01). The high dose of XSC can inhibit P-selection level markedly (*P* < 0.05), this has similar effect with the Aspirin group (see Figure 1).

### 3.4. XSC Reduces the Platelet Gelsolin Level and Enhances the Activity of Extracellular Actin-Scavenger System (EASS)

**Figure 2:**
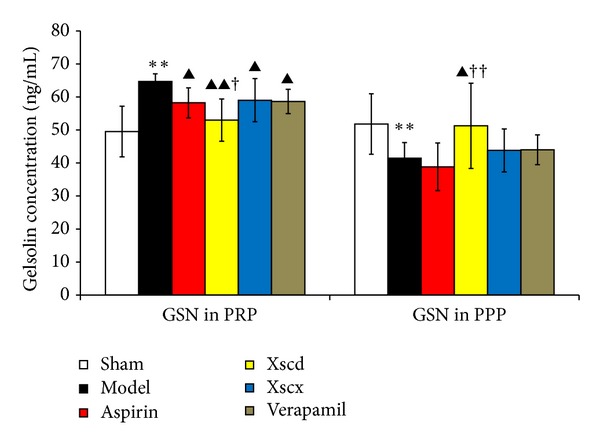
Effect of Xiongshao Capsule (XSC) on Gelsolin concentration among PRP and PPP of AMI rats. **P < 0.01 compared to Sham group, ^▴^ *P* < 0.05 or ^▴▴^ *P* < 0.01 compared to Model group, and ^†^ *P* < 0.05 or ^††^ *P* < 0.01 compared to Aspirin group.

**Figure 3:**
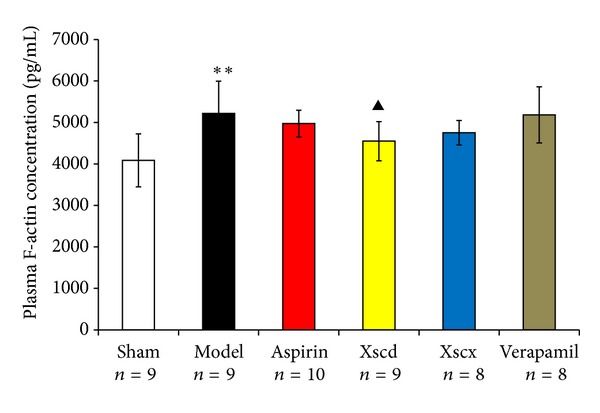
Effect of Xiongshao Capsule (XSC) on Plasma F-actin concentration of AMI rats. **P < 0.01 compared to Sham group, and ^▴^ *P* < 0.05 compared to Model group.

**Figure 4:**
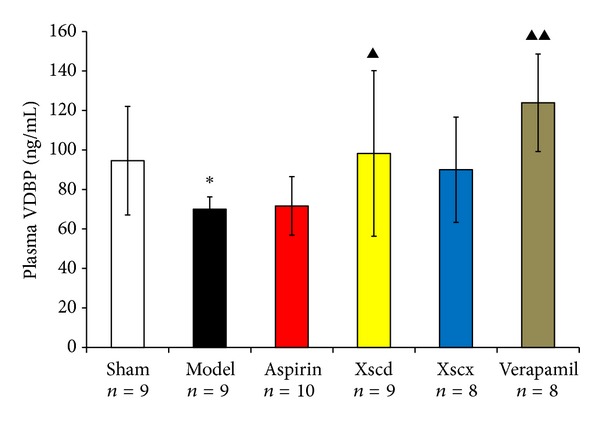
Effect of Xiongshao Capsule (XSC) on Plasma VDBP concentration of AMI rats. *P < 0.05 compared to Sham group, and ^▴^ *P* < 0.05 or ^▴▴^ *P* < 0.01 compared to Model group.

Plasma gelsolin and VDBP are the main components of the EASS which undertake the responsibility as scavenger of the abnormal increased extracellular filament actin (F-actin). Compared with the Sham group, the plasma gelsolin and VDBP of the Model group was reduced significantly (*P* < 0.05) and F-actin increased markedly (*P* < 0.01), while platelet gelsolin it increased markedly (*P* < 0.01). High dose of XSC can reduce platelet gelsolin and F-actin level (*P* < 0.05), while it increased plasma gelsolin and VDBP significantly (*P* < 0.05) (see Figures 2, 3, and 4).

### 3.5. XSC Inhibits the Activation of TXB_2_ and COX-1

**Figure 5:**
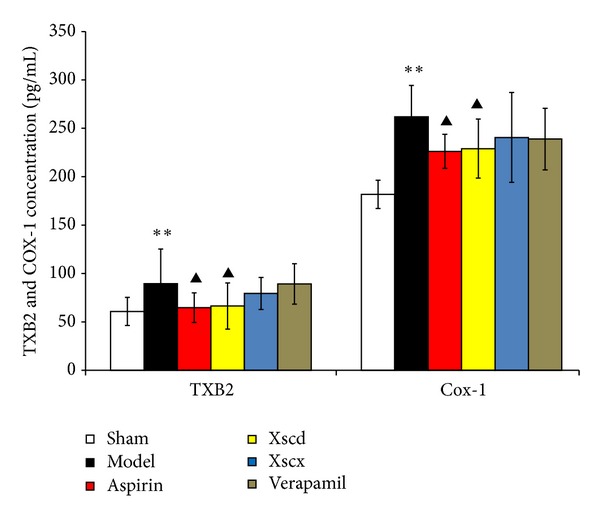
Effect of Xiongshao Capsule (XSC) on Plasma TXB_2_ and COX-1 concentration of AMI rats. **P < 0.01 compared to Sham group, and ^▴^ *P* < 0.05 compared to Model group.

Compared with Sham group, the concentration of TXB_2_ and COX-1 of Model group increased significantly after ligation of LAD for 3 hours (*P* < 0.01). High dose of XSC can reduce COX-1 and TXB_2_ level significantly (*P* < 0.05); this has similar effect with the Aspirin group (see Figure 5).

### 3.6. XSC Inhibits the MFI of Calcium

**Figure 6:**
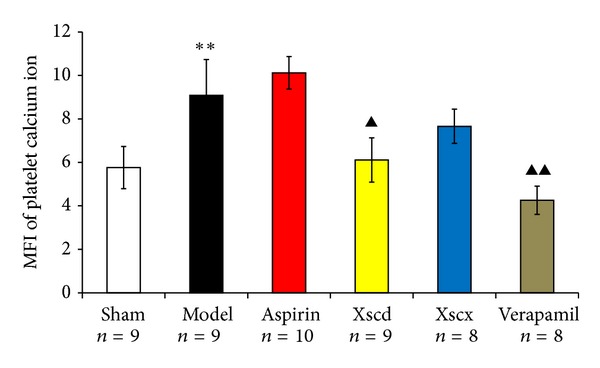
Effect of Xiongshao Capsule (XSC) on MFI of platelet calcium ion concentration of AMI rats. **P < 0.01 compared to Sham group, and ^▴^ *P* < 0.05 or ^▴▴^ *P* < 0.01 compared to Model group.

Compared with Sham group, the MFI of calcium of the Model group increased markedly (*P* < 0.01), High dose of XSC can inhibit platelet calcium increase (*P* < 0.05). This has similar effect to the Verapamil group (*P* < 0.05) (see Figure 6).

### 3.7. XSC Attenuates the Expression of Gelsolin in Infarcted Myocardium

**Figure 7:**
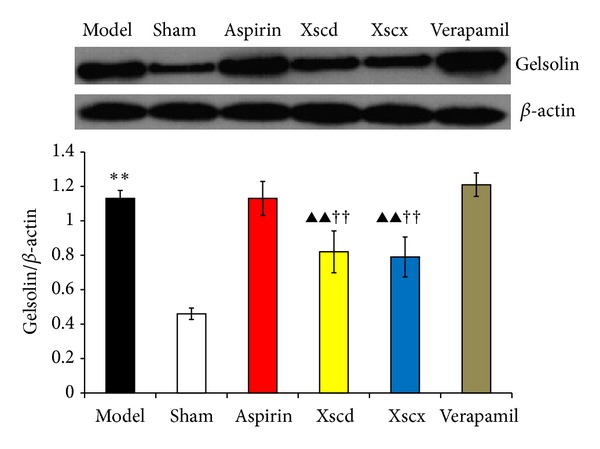
Effect of Xiongshao Capsule (XSC) on protein level of gelsolin in ischemic heart tissue of AMI rats. **P < 0.01 compared to Sham group, and ^▴▴^ *P* < 0.01 compared to Model group, ^††^ *P* < 0.01 compared to Aspirin group.

Compared with Sham group, the gelsolin expression of infarcted myocardium of Model group increased markedly, and XSC can inhibit gelsolin expression of infarcted myocardium, but verapamil has no such effect (see Figure 7).

### 3.8. Analyses of Correlation between Platelet Gelsolin Concentration and Plasma P-Selectin Level

**Figure 8:**
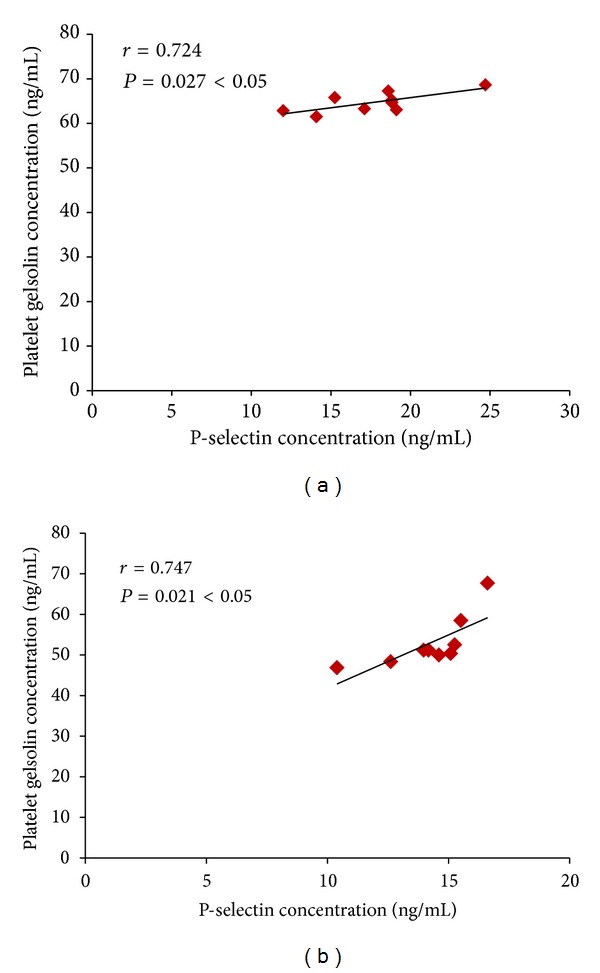
Correlation between gelsolin concentration in PRP and plasma P-selectin level of Model group and Xscd group. (a) Model group and (b) Xscd group.

Next we investigated any potential correlation between the platelet gelsolin concentration and plasma P-selectin levels that may exist in the Model group and Xscd group. Correlation analysis showed that platelet gelsolin concentrations were high positively correlated with plasma P-selectin levels in the Model group (see Figure 8(a)) and Xscd group (see Figure 8(b)).

## 4. Discussion

Gelsolin is a calcium-regulated actin filament (F-actin) severing and capping protein, which is expressed as both cytoplasmic and plasma isoforms. The functions of extracellular gelsolin are less well defined. Gelsolin is also an important cytoskeletal protein, which is a key actin binding protein (ABPs) as well. Increasingly evidence has shown that gelsolin has close relationship with many diseases and pathological processes, such as cancer, apoptosis, infection and inflammation, pulmonary diseases, and aging [21]. During the past 5 years, many scholars began to focus on gelsolin's possible role in the development of cardiovascular diseases [22]. Activated platelets play a pivotal role in the formation of arterial thrombi, and antiplatelet drugs become the core in the prevention and treatment of CVD. Platelet activation not only causes the changes of membrane protein, but also a series of morphological changes, from inviscid, discotic circulating platelets to a paste-like, protruding platelet jelly, that affects the regulation of platelet cytoskeletal proteins. 

Using differential proteomics of platelet, our previous study [9] indicated that platelet gelsolin was the main platelet differential functional proteins between patients with coronary heart disease and healthy people. In addition, data from our previous clinical studies demonstrated that [10, 11] platelet gelsolin was highly expressed in patients with acute coronary syndrome (ACS) and the blood stasis syndrome (BSS) of traditional Chinese medicine. Meanwhile, based on the Chinese medicine principle of “prescription to syndrome.” Platelet gelsolin may be viewed as a new target for ABC herbs. In this study, we evaluated the biological role of platelet gelsolin in the development of platelet activation in a rat model of AMI and the potential contribution of XSC prophylaxis in this progress *in vivo*.

As we know, P-selectin is a 140 kD glycoprotein that is presented in the granules of platelets and translocates rapidly to the cell surface after platelet activation; it is generally considered as the gold marker of platelet activation [23]. In this study, after ligation of LAD for 3 hours, the concentration of CK-MB and cTnI and the P-selectin level of the Model rats increased significantly compared with Sham rats, which indicated that the model rats had myocardial injury and platelet activation.

The actin cytoskeleton plays a central role in many fundamental cellular processes involving the generation of force and facilitation of movement, which are enabled by the assembly of actin monomers into filaments and cooperation with a wide variety of ABPs [21], including gelsolin. Actin monomers (G-actin) spontaneously associates to form F-actin under physiological conditions and vice versa. This dynamic progress keeps in balance all the time. In the presence of tissue injury or cell death, G-actin is released into the circulation where it can interact with components of the haemostatic and fibrinolytic systems, or polymerize and form F-actin excessively. Studies [24] have suggested that F-actin can lead to platelet aggregation directly *in vitro*, and the presence of excessive F-actin in blood vessels, which can plug smaller vessels and decrease blood flow to promote the formation of blood clots, can be fatal. Infusion of high doses of G-actin in rabbits caused the rapid and fatal formation of massive F-actin-containing thrombi in arterioles and capillaries of pulmonary veins [25]. An extracellular actin-scavenger system (EASS) [12] was therefore likely to exist. Plasma gelsolin, together with vitamin D binding protein (VDBP), another extracellular ABPs, were regarded as potentially important components of this system. They are capable of removing F-actin from the circulation and inhibiting F-actin elongation to alleviate the vascular toxicity of excessive F-actin. In this study, the concentration of gelsolin in PRP of AMI rats increased accompanied by the high platelet activation and increased level of F-actin while gelsolin in PPP decreased which indicates the EASS of AMI rats was suppressed. Correlation analysis showed that platelet gelsolin concentrations were high, positively correlated with plasma P-selectin levels in the Model group.

Xiongshao capsule (XSC) is a patent drug developed from Xue Fu Zhu Yu Decoction. It is the classic formula used for activating blood circulation (ABC) in China for hundreds of years. Clinical studies have shown that XSC can effectively prevent restenosis after percutaneous coronary intervention (PCI) [17]. XSC was shown to enhance the protective effect of ischemic postconditioning on rat with myocardial ischemic reperfusion injury [26]. It was also shown to stabilize atherosclerotic plaque by suppressing inflammation and the expression of Fc*γ*RIIIA [27]. But the potential antiplatelet mechanism of XSC prophylaxis is unclear. In this study, we found that high dose of XSC prophylaxis could decrease the concentration of myocardial injury markers, CK-MB and cTnI, and reduce the plasma P-selectin level of AMI rats as well. The antiplatelet mechanism of aspirin involves the inhibition of COX-1 and TXA_2_, our study shows that high dosage of XSC can inhibit the activation of TXB_2_ and COX-1 *in vivo*, which has similar cardioprotection effect with aspirin *in vivo*. Meanwhile, high dosage of XSC prophylaxis inhibited the expression of platelet gelsolin in AMI rats by inhibiting the platelet calcium influx, but increased the concentration of plasma gelsolin and plasma VDBP simultaneously, so the EASS was activated, and the concentration of F-actin in AMI rats decreased which indicated that the F-actin was being removed from the circulation. Calcium ions not only promote gelsolin secretion but also play a vital role in the development of platelet activation. Studies have shown increased platelet [Ca^2+^]_*i*_ in patients with CVD [28], and that calcium channel blocker (CCB) could reduce platelet [Ca^2+^]_*i*_ and inhibit platelet aggregation [29]. Verapamil is a classic CCB agent and a previous study *in vivo* [30] shows that verapamil exhibits a dose-dependent inhibitory effect on platelet aggregation and thrombus formation in rats. In this study, our results show that high dosage of XSC can mimic the calcium channel antagonist effect. 

We have also investigated the expression of the gelsolin in infarcted myocardium of AMI rats. The results indicate that the gelsolin expression of infarcted myocardium of the Model group increased markedly; while XSC can inhibit gelsolin expression of infarcted myocardium significantly, verapamil or aspirin has no such effect, holding that other pathway existed in the regulation of gelsolin as well. Heart failure (HF) is the end stage of CVD (including after AMI). It is of great importance to know the effects and mechanism of XSC on cardioprotection at earlier stages of CVD. Ventricular remodeling after AMI is the main pathological change of HF. A previous study [31] has showed that gelsolin is an important contributor to heart failure progression through novel mechanisms of HIF-1*α* and DNase I activation and downregulation of antiapoptotic survival factors. Based on these results and our study, we propose that gelsolin inhibition is a promising target for CVD therapy besides antiplatelet agent.

## 5. Conclusion

We have provided experimental evidence supporting our conclusion that high correlation between platelet gelsolin and platelet activation level in AMI rats, the aspirin-like cardio-protection, and antiplatelet effects of XSC are related to its inhibition on platelet gelsolin, platelet calcium influx and activated the EASS. Taken together, our results suggest that platelet gelsolin is a potential antiplatelet target and XSC is a promising lead compound for antiplatelet and cardiovascular therapy.

## Figures and Tables

**Table 1:** The outcome among the different groups after ligation of LAD.

Group	*N*	Dead rats (*n*)	Surviving rats (*n*)
Sham	15	6	9
Model	15	6	9
Aspirin	15	5	10
Xscd	15	6	9
Xscx	15	7	8
Verapamil	15	7	8

**Table 2:** Effect of Xiongshao Capsule (XSC) on the concentration of myocardial injury markers of AMI rats.

Group	*N*	CK-MB (ng/mL)	cTnI (pg/mL)
Sham	9	0.279 ± 0.074	9.81 ± 2.62
Model	9	0.386 ± 0.043**	15.18 ± 4.3**
Aspirin	10	0.340 ± 0.024^†^	12.04 ± 1.19^†^
Xscd	9	0.336 ± 0.027^†^	12.23 ± 1.41^†^
Xscx	8	0.351 ± 0.013	13.85 ± 3.02
Verapamil	8	0.358 ± 0.017	14.43 ± 2.98

**P < 0.01 compared to Sham group and ^†^ *P* < 0.05 compared to Model group.

## References

[1] Choi J, Kermode JC (2011). New therapeutic approaches to combat arterial thrombosis. *Molecular Interventions*.

[2] Tseeng S, Arora R (2008). Reviews: aspirin resistance: biological and clinical implications. *Journal of Cardiovascular Pharmacology and Therapeutics*.

[3] Michelson AD (2010). Antiplatelet therapies for the treatment of cardiovascular disease. *Nature Reviews Drug Discovery*.

[4] Baigent C, Blackwell L, Collins R (2009). Aspirin in the primary and secondary prevention of vascular disease: collaborative meta-analysis of individual participant data from randomised trials. *The Lancet*.

[5] Juurlink DN, Gomes T, Ko DT (2009). A population-based study of the drug interaction between proton pump inhibitors and clopidogrel. *Canadian Medical Association Journal*.

[6] Mega JL, Close SL, Wiviott SD (2009). Cytochrome P-450 polymorphisms and response to clopidogrel. *New England Journal of Medicine*.

[7] Seshasai SR, Wijesuriya S, Sivakumaran R (2012). Effect of aspirin on vascular and nonvascular outcomes: meta-analysis of randomized controlled trials. *Archives of Internal Medicine*.

[8] García A (2010). Clinical proteomics in platelet research: challenges ahead. *Journal of Thrombosis and Haemostasis*.

[9] Li XF, Jiang YR, Wu CF, Chen KJ, Yin HJ (2009). Study on the correlation between platelet function proteins and symptom complex in coronary heart disease. *Zhongguo Fen Zi Xin Zang Bing Xue Za Zhi*.

[10] Liu Y, Yin HJ, Chen KJ (2011). Research on the correlation between platelet gelsolin and blood-stasis syndrome of coronary heart disease. *Chinese Journal of Integrative Medicine*.

[11] Liu Y, Yin HJ, Jiang YR, Xue M, Chen KJ (2012). Correlation between platelet gelsolin level and different types of coronary heart disease. *Chinese Science Bulletin*.

[12] Lee WM, Galbraith RM (1992). The extracellular actin-scavenger system and actin toxicity. *New England Journal of Medicine*.

[13] Chen KJ (2008). Explore the possibilities of Chinese herb and formulas for promoting blood circulation and removing blood stasis on reducing the cardiovascular risk. *Zhongguo Zhong Xi Yi Jie He Za Zhi*.

[14] Liu Y, Yin HJ, Shi DZ, Chen K-J (2012). Chinese herb and formulas for promoting blood circulation and removing blood stasis and antiplatelet therapies. *Evidence-Based Complementary and Alternative Medicine*.

[15] Zhang Z, Qing LM, Chen KJ (2000). Study on the pharmacokinetics of paeoniflorin contained in Xiongshao capsule in canine. *Zhongguo Shi Yan Fang Ji Xue Za Zhi*.

[16] Zhang Z, Yan YF, Chen KJ (2001). Study on the pharmacokinetics of ferulic acid in canine serum after giving an intragastrical single dose of Xiongshao capsules to a dog. *Beijing Zhong Yi Yao Da Xue Xue Bao*.

[17] Chen KJ, Shi DZ, Xu H (2006). XS0601 reduces the incidence of restenosis: a prospective study of 335 patients undergoing percutaneous coronary intervention in China. *Chinese Medical Journal*.

[18] Sun M, Dawood F, Wen WH (2004). Excessive tumor necrosis factor activation after infarction contributes to susceptibility of myocardial rupture and left ventricular dysfunction. *Circulation*.

[19] Li GH, Shi Y, Chen Y (2009). Gelsolin regulates cardiac remodeling after myocardial infarction through DNase I-mediated apoptosis. *Circulation Research*.

[20] M M, Zhuang, Wen YX, Liu SL (2005). Determination of the level of cytopplasmic free calcium in human platelets with flow cytometry. *Xi An Jiao Tong Da Xue Xue Bao*.

[21] Li GH, Arora PD, Chen Y, McCulloch CA, Liu P (2012). Multifunctional roles of gelsolin in health and diseases. *Medicinal Research Reviews*.

[22] Liu Y, Jiang YR, Yin HJ (2011). Gelsolin and cardiovascular diseases. *Zhongguo Fen Zi Xin Zang Bing Xue Za Zhi*.

[23] Michelson AD, Furman MI (1999). Laboratory markers of platelet activation and their clinical significance. *Current Opinion in Hematology*.

[24] Vasconcellos CA, Lind SE (1993). Coordinated inhibition of actin-induced platelet aggregation by plasma gelsolin and vitamin D-binding protein. *Blood*.

[25] Haddad JG, Harper KD, Guoth M (1990). Angiopathic consequences of saturating the plasma scavenger system for actin. *Proceedings of the National Academy of Sciences of the United States of America*.

[26] Zhang DW, Zhang L, Liu JG (2010). Effects of Xiongshao capsule combined with ischemic postconditioning on monocyte chemoattractant protein-1 and tumor necrosis factor-*α* in rat myocardium with ischemic reperfusion injury. *Zhongguo Zhong Xi Yi Jie He Za Zhi*.

[27] Huang Y, Yin HJ, Ma XJ (2011). Correlation between Fc*γ*RIIIA and aortic atherosclerotic plaque destabilization in ApoE knockout mice and intervention effects of effective components of Chuanxiong Rhizome and Red Peony Root. *Chinese Journal of Integrative Medicine*.

[28] Yoshimura M, Oshima T, Hiraga H (1998). Increased cytosolic free Mg^2+^ and Ca^2+^ in platelets of patients with vasospastic angina. *American Journal of Physiology*.

[29] Fujinishi A, Takahara K, Ohba C, Nakashima Y, Kuroiwa A (1997). Effects of nisoldipine on cytosolic calcium, platelet aggregation, and coagulation/fibrinolysis in patients with coronary artery disease. *Angiology*.

[30] Li W, Liu Y, Huang Y, Ji Y (2007). Effect of verapamil on the thrombogenes is and nitric oxide level in the serum of rats. *Nanjing Yi Ke Da Xue Xue Bao*.

[31] Li GH, Shi Y, Chen Y (2009). Gelsolin regulates cardiac remodeling after myocardial infarction through DNase I-mediated apoptosis. *Circulation Research*.

